# Changes in consumers’ awareness and interest in cosmetic products during the pandemic

**DOI:** 10.1186/s40691-021-00271-8

**Published:** 2022-01-05

**Authors:** Yeong-Hyeon Choi, Seong Eun Kim, Kyu-Hye Lee

**Affiliations:** 1 Dept. of Clothing and Textiles, Hanyang University, 222 Wangsimni-ro, Seongdong-gu, Seoul, Korea; 2 Department of Textile and Apparel Management, University of Missouri, Columbia, Missouri USA

**Keywords:** Pandemic, COVID-19, Beauty awareness, Maskne, Google Trends, Social network analysis

## Abstract

This research investigates the impact of the COVID-19 pandemic on consumers’ perspectives of beauty and individual cosmetic products. Since the first confirmed case of COVID-19 was announced on December 31st, 2019, the search volumes of Google News have been updated and information on confirmed cases of the disease has been collected. This study used Python 3.7, NodeXL 1.0.1, and Smart PLS 3.0 to analyze consumer awareness of cosmetic products during the pandemic. The results reveal that consumers’ perspectives of beauty are impacted by a pandemic. Global consumers perceive skincare as an important aspect during the pandemic, while the importance of makeup fell after the outbreak. The awareness of skincare and makeup products has changed. The spread of the pandemic (SOP) has a positive impact on skincare products, but a negative impact on makeup products, except for eye makeup products, which was positive. Finally, the SOP was not significant in terms of consumers’ interest in masks. Fifth, interest in masks showed a positive relationship with interest in skincare products, such as cleansing products, while a negative relationship was observed with interest in makeup products. Overall, this study concludes that pandemics certainly have an impact on global consumers’ perspectives. As a pandemic spread, interest in skincare products increases, while interest in makeup products decreases. This study has academic significance in that it investigates the effects of consumption of cosmetic products during the stay-at-home rules. It can be used as standard information for setting marketing strategies in pandemic-like situations in the future.

## Introduction

The coronavirus disease‑19 (COVID‑19) is an infectious disease caused by the severe acute respiratory syndrome coronavirus‑2 (SARS-CoV‑2) (Babu, 2020). As the number of confirmed cases of COVID-19 increased, the World Health Organization (WHO) announced a pandemic on March 11th, 2020. In an attempt to slow the pandemic, the WHO announced personal hygiene guidelines. Governments all over the world have induced people to follow the guidelines, which include regularly washing hands, social distancing, and wearing a medical mask (Cartaud et al., 2020). As the period of social distancing has been prolonged and people are wearing personal protective equipment (PPE) to prevent themselves from being infected, the overall demand for cosmetics has declined (Wischhover, 2020).

Since consumers now have to stay in their homes longer, they are stressed over difficulties in terms of their appearance (Pikoos et al., 2020). This situation led to people feeling less need or opportunity to wear makeup; hence, the overall demand for makeup products has dropped (Biskanaki et al., 2020). However, not all signals for the cosmetic industry are negative. Due to the current situation being conducive to wearing masks for prolonged periods, consumers are suffering from acne, and they are focusing more on skincare products (Rubin, 2020; Schiffer, 2020). Although the interest in makeup products has decreased, that for skincare products has shown a different direction in terms of sales. Interestingly, this behavior is a global phenomenon. Given that the preference for makeup products, such as eyeshadows or lipstick, is different from beauty awareness in each country, the present skincare-focused phenomena follow similar reactions and patterns. This indicates that a pandemic-like situation can make consumer’s attitude toward cosmetic products somewhat different to what it was before such a situation.

This behavior raises another intriguing point. The motivation of applying makeup is to express one’s identity, gain confidence, or be seen as a well-mannered person (Karabulut et al., 2020). No matter how little makeup consumers wear due to the PPE and stay-home policy, they still engage in social interactions. If consumers perceive wearing PPE as a tool for hiding themselves, they would not have an intention to use certain products. On the other hand, if consumers are only focused on survival during the pandemic, the results of cosmetic behavior would be more complicated.

Our research goal is to determine consumers’ cosmetic behavior in the COVID-19 pandemic situation. To do so, we (1) compare the perceptions of cosmetic products before and after the outbreak of COVID-19 using social big data analysis. (2) We establish the causal relationship between COVID-19 variances and purchase behavior of cosmetic products using partial least squares structural equation modeling (PLS-SEM). From a macro perspective, this study reveals the impact of COVID-19 on consumers’ attitude toward cosmetic and skincare products. The multi-dimensional approaches used herein contribute to an in-depth understanding of consumer behavior in the COVID-19 situation.

## Literature review

The social self is “a tug-of-war” between societal similarity and individual uniqueness (Brewer, 1991). It is a human need to conform with others while simultaneously maintaining their individual presence. There are various ways to reveal one’s social self to society. Of these, makeup is a method to reveal and express one’s social self to society (Lee & Oh, 2018). In addition, according to which shades and the degree of makeup (from bare face to full makeup) a person wears, their social appearance could be easily and instantly changed. Due to this salience of makeup, the degree of makeup status is not only someone’s style but a metaphor for their feelings or intentions. However, COVID-19 has forced everyone to wear masks, which is a critical obstacle in constructing a social self through makeup. Thus, the decision of whether to apply makeup is an important indicator of cosmetic behavior, perception of a pandemic situation, and maintenance of societal appearance.

### Social self and maskne

In the past, makeup was considered the preserve of women (Cash & Cash, 1982; Miller & Cox, 1982). However, male consumers have recently begun to show increased interest in caring for their appearances, which has led to a growth in the male cosmetic products market (Iida, 2006). Generation Y men tend to express their self-esteem through fashion products and are concerned about their bodies and weight (Sung & Yan, 2020). This phenomenon could provide the reason why male consumers have an increased interest in makeup, which is commonly viewed as an aesthetic concept. In fact, men want to be seen as more charming and masculine, not feminine (Souiden & Diagne, 2009). Thus, the context of makeup has shifted from being seen as more feminine to being able to express oneself to society (Kim & Choi, 2020).

There is now a dilemma regarding using makeup to reveal one’s social self. Due to COVID-19, wearing a mask is a normal state or mandatory regulation in some countries when people go out, meet someone, or are in a public space. This regulation obliges people to cover half of their facial area, which has induced consumers to practice a passive attitude toward cosmetics (Han et al., 2020). The fact that masks can cause flare-ups of acne is considered one of the main reasons for this attitude. As the inner temperature of the mask causes flare-up in the facial area, many consumers have suffered from acne due to prolonged periods of wearing masks (Park et al., 2020). Given the significantly positive correlation between cosmetics and acne (Perera et al., 2018), the sealing effect of the mask makes the wearer more susceptible to acne flare-ups. It is popularly known as “maskne or mask acne” (Kosasih, 2020).

The dilemma of makeup starts here. To some, applying makeup could be a really important ritual that helps them prepare to present their social self. However, due to the COVID-19 pandemic, there is an adverse synergy between makeup and wearing a mask for an extended period due to the aforementioned side effects, like acne or flushing (Yu, 2020). It presents an awkward situation in which wearing makeup causes bad skin conditions and going bare faced is somewhat embarrassing. If someone decides to wear makeup for social activity, they make it their priority to maintain their social self. On the other hand, if someone does not apply makeup when they are going out, they put more weight on caring for their skin conditions. The side effects of wearing a mask for long periods could be considered a minor problem when the benefit is maintaining our health. Given that makeup is one of the ways to present a good social self, a no-makeup situation may induce feelings of shame. In particular, consumers who are very aware of their surroundings and others’ evaluation may regard going bare faced as a shameful situation. It leads to degradation of their social self-esteem and also increases stress (Gruenewald et al., 2004).

In a pandemic-like situation, people experience death, anxiety, and depression (Lee et al., 2020). Anxiety regarding appearance management occurs. Pikoos et al. (2020) revealed that during COVID-19 restrictions, societal appearance pressure persisted in individuals with high dysmorphic concerns. Pikoos et al. (2020) found that a highly appearance-concerned group displayed feelings of pain and suffering due to the shutdown of beauty services, and the demand for cosmetics once restrictions are lifted has increased. This is the result of the anxiety that they cannot maintain their appearance and worry about it when they go out. This study regards pandemic anxiety variance as the number of confirmed cases. It means that as more confirmed cases occur and COVID-19 spreads, this leads to higher levels of anxiety (Elbay et al., 2020). Therefore, it is expected that if the spread of the pandemic (SOP) becomes more severe, anxiety will increase. As a result, the interest in all cosmetic product categories (skincare and makeup products) would show an incremental relationship.

- **Research question 1 (R1):** Pandemics will change consumers’ awareness of cosmetic products (skincare and makeup).
- **Hypothesis 1 (H1): **Spread of the pandemic (SOP) will positively influence interest in skincare products.
- **Hypothesis 2 (H2): **Spread of the pandemic (SOP) will positively influence interest in makeup products.

As one of the preventive measures, wearing a mask has become a default option during outdoor activities. In the early phase of the pandemic, there was significant interest in wearing a mask, as it was not a default option. However, before the outbreak of COVID-19, it was considered unusual behavior. More than a year since the outbreak began, mask wearing is no longer considered abnormal or special behavior. In a pandemic situation, the period during which a disease remains contagious is long time, and the interest in preventive measures shows a declining tendency (Bults et al., 2011). Consumers concentrate on their discomfort. In particular, wearing a mask for prolonged durations causes maskne, which is one of the direct and immediate side effects of the activity. This study expects that the interest in masks leads to incremental interest in skincare products but not in makeup products.

- **Hypothesis 3 (H3): **Spread of the pandemic (SOP) will not influence interest in face masks.
- **Hypothesis 4 (H4): **Interest in face masks will positively influence interest in skincare products.
- **Hypothesis 5 (H5): **Interest in face masks will negatively influence interest in makeup products.

**Fig. 1:**
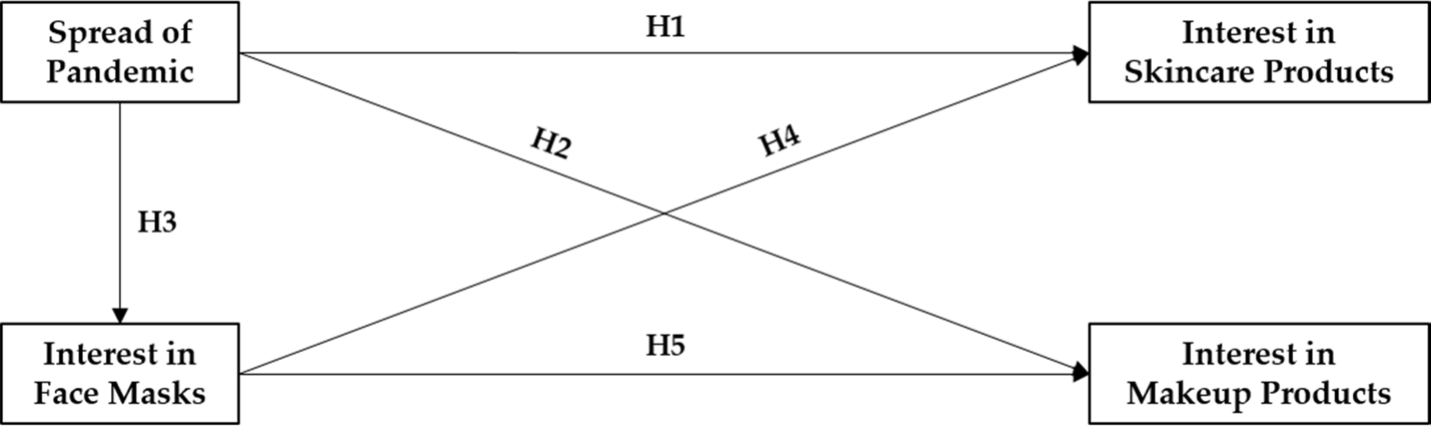
Conceptual research framework

The main variables and hypotheses of this study are illustrated in Fig. 1.

## Methods

### Social network analysis

One of the characteristics of social network and big data analysis is being able to investigate the potentially key elements of a study. This is done by extracting essential data and analyzing it through the bottom-up approach. It is cost effective and consumes less time as it does not require surveys or interviews to be conducted and this method is now being used in various fields (Niyirora & Argones, 2019; Wu et al., 2017). Social network analysis, the most commonly used method of analyzing big data, deduces the characteristics of the network by determining occurrence and regularity through appearance frequency and relationships between words (Park & Leydesdorff, 2004). Centrality, a measure of the central location of a node within a network, is used in the interpretation of data (Hanneman & Riddle, 2005).

Ahmed et al. (2020) investigated the COVID-19 situation using social network analysis. The authors examined Twitter posts and investigated the motive of the 5G COVID-19 conspiracy theory. They confirmed that the most popular web sources shared by users are fake news websites. Based on Twitter posts, Yum (2020) analyzed the role of public and political administrators in COVID-19 pandemic situation using social network analysis. Choi and Lee (2020) used text mining and social network analysis to examine the changes in consumers’ perspectives of fashion products during the pandemic. Korean fashion consumers were mainly concerned with precautions, home life, digital and cosmetic products, online channels, and consumption. This indicates a stronger stance on precaution than fashion during COVID-19 than in previous pandemics (SARS, MERS).

### Measurement: Tf-idf and centralities

Term frequency-inverse document frequency (*Tf-idf*) and central value were measured and then analyzed. In PLS-SEM, development of worldwide confirmed cases was used as an exogenous variable, and search volumes of individual cosmetic products was used as an endogenous variable. Python 3.7 was used to measure *Tf-idf* value, and NodeXL 1.0.1 was used in the network analysis stage to measure centrality. *Tf-idf*, a measuring standard that supplements the frequency, is normally used to judge the importance of a certain word in a corpus. *Tf-idf* weighting is *term frequency (tf)* $\times$ *inverse document frequency (idf)*. Given a collection of terms *t* $\in$ *T* that appear in a set of *N* documents *d* $\in$ *D*, each of length *n*_*d*_, *Tf-idf,* weighting is computed as shown in Eq. 1 (Choi & Lee, 2021; Yahav et al., 2019).

Centrality analysis defines the meanings of each keyword in the context of a document (Choi et al., 2021), which are indicated in terms of degree, betweenness, closeness, and eigenvector centrality (Kwahk, 2014), as shown in Eqs. 2–5. The centrality measure can be summarized by standardizing the equation as follows: (1) ${C}_{x}({N}_{i})$ is the centrality of actor *i* in each calculation. (2) Every *g* is the number of actor *i* in networks (Kwahk, 2014; Wasserman & Faust, 1994). (3) The standardized actor betweenness centrality is then divided by the maximum value ((*g* − 1)(*g* − 2)/2) of the theoretical betweenness centrality (Freeman, 1979; Wasserman & Faust, 1994). (4) The value of closeness centrality for the number of actors *g* is ${C}_{c}({N}_{i})$=1/(*g* − 1), and the standardized actor closeness centrality is multiplied by *g* − 1 to consider the size of the entire network (Beauchamp, 1965; Freeman, 1978). (5) In eigenvector centrality, actor *i* is the *i*th element of unit eigenvector *e*, and *e* represents the largest eigenvalue of the adjacency matrix, with *x* as an element. *X* is an adjacency matrix with ${X}_{ij}$ as an element, and $\lambda$ is an array of eigen values (Bonacich, 2007; Kwahk, 2014). To obtain eigenvector centrality, multiple steps of computation are performed. In the first step, the centrality value is calculated as the sum of the degree for *g*. The centrality value of each actor is then calculated as the sum of the first-stage centrality values of each actor. The final centrality values are calculated as the sum of the results of the second stage. This step-by-step computation process is repeated until the centrality value no longer changes.

1 $$\begin{gathered} tf_{{t,d}} = \,\frac{{f_{{t,d}} }}{{n_{d} }}\,\,idf_{t} = \,\log \frac{N}{{df_{1} }} \hfill \\ W_{{t,d}} = \,tf_{{t,d}} \times \,idf_{t} \hfill \\ \end{gathered}$$

Equation 1 Calculation of *Tf-idf*

2 $$C'_{D} \,\left( {N_{i} } \right)\, = \,\frac{{C_{D} \left( {N_{i} } \right)}}{{g - 1}}$$

Equation 2 Degree centrality of actor *i*

3 $$C'_{B} \left( {N_{i} } \right)\, = \,\frac{{C_{B} \left( {N_{i} } \right) \times 2}}{{\left( {g - 1} \right)\left( {g - 2} \right)}}$$

Equation 3 Betweenness centrality of actor *i*

4 $$C'_{c} \left( {N_{i} } \right) = \,\left( {g - 1} \right)\left( {C_{c} \left( {N_{i} } \right)} \right)$$

Equation 4 Closeness centrality of actor *i*

5 $$C_{E} \left( {N_{i} } \right) = \,\lambda \sum\limits_{j}^{g} {x_{{ij}} } C_{E} \left( {N_{j} } \right),\,i \ne j$$

Equation 5 Eigenvector centrality of actor *i.*

### Preliminary investigation

The occurrence of the first recorded case of COVID-19 on December 31st, 2019 until September 30th 2020 was set as the standard period for collecting data. To compare consumers’ awareness, data between December 31st, 2018 and September 30th, 2019 were also collected. Preliminary investigation was conducted before selecting keywords to be investigated. We analyzed Google News, Twitter posts, and YouTube comments containing the words “corona” and “beauty” for nine months following the outbreak of COVID-19 (total: 8192).

As a result of preliminary investigation, according to appearance frequency, “mask” (687), “skincare” (490), “makeup” (459), and “haircare” (330) were identified as the beauty keywords related to COVID-19. In the network analysis, “skincare,” “makeup,” and “beauty” were set as the keywords. “Mask” showed a relationship with “corona,” but it also showed a strong relationship with “face” and “facial.” This suggests it may relate to medical masks and skin management masks, so it was excluded from the analysis. Google News was selected as the collecting channel as the data contained relatively less noise (Table 1).

**Table 1:** Volume of data for each keyword

	Beauty	Skincare	Makeup
Before COVID-19 outbreak: 2018.12.31–2019.9.30	362	344	300
Before COVID-19 outbreak: 2019.12.31–2020.9.30	326	356	325

All data were collected from Google News

### Data collection and analysis for PLS-SEM

PLS-SEM analysis was used to identify the impact of the spread of infectious disease on consumers’ interest in cosmetic products. To determine the development of the spread, worldwide confirmed cases were collected from the conoraboard.kr website, which provides real-time information about COVID-19. The data collection period was from January 21st, 2020 to September 30th, 2020, the range provided by the website. Consumers’ interest in cosmetic products is based on data on search volumes for keywords in Google Trends. The data were collected globally and limited to the category of “beauty & fitness.” In contrast, previous studies use “mask” as the main beauty keyword (e.g. Choi & Lee, 2020). Therefore, we identified the search volume data of the keywords “mask” and “acne.” Sub products of the specific cosmetic products are set according to social big data analysis in the *Tf-idf* standard.

To verify the model, we used development of coronavirus confirmed cases and search volume of keywords in Google Trends. Spread of disease is defined as the development of coronavirus confirmed cases. The measurement tool is the number of daily confirmed cases from January 21st to September 30th, 2020 obtained from conoraboard.kr. Mask interest is defined as consumers’ interest in masks, measured by the search volume of “mask” in Google. Interest in skincare and makeup are defined as consumers’ interest in individual products, measured by the search volume of all relative keywords in Google. As per the results of social network analysis, keywords with high *Tf-idf* are selected as representative items. Data on representative keywords are also collected within the same amount of time, using the same methods. A total of 253 records were collected and 13 items were measured. Smart PLS 3.0 was used to determine the influencing relationship. In PLS-SEM, the sampling was conducted 5000 times via bootstrapping. After verifying path coefficients and significance, it was evaluated by identifying the *R*^*2*^, *f*^*2*^, and *Q*^*2*^ values.

## Results and discussion

### Comparison of consumers’ beauty awareness in the pandemic

#### Based on Tf-idf value

Since the announcement of the first confirmed case of coronavirus on December 31st, 2019, social data including the keyword “beauty” have been collected. Keywords were distilled and centrality score and *Tf-idf*, which can complement simple frequency, were measured. The top 50 keywords were used in the analysis and the top 30 keywords according to the *Tf-idf* are reported in Table 2.

**Table 2:** Consumers’ cosmetic awareness before and after COVID-19 outbreak

No	Before COVID-19 outbreak						After COVID-19 outbreak					
	Word	***Tf-idf***	C_d_^a^	C_b_^b^	C_c_^c^	C_e_^d^	Word	***Tf-idf***	C_d_^a^	C_b_^b^	C_c_^c^	C_e_^d^
1	Makeup	369.68	0.84	258.70	0.02	0.05	Product	410.57	0.76	134.44	0.02	0.04
2	Product	315.68	0.73	150.32	0.02	0.05	Makeup	389.54	0.78	154.22	0.02	0.05
3	Brand	276.77	0.57	56.08	0.01	0.04	Brand	328.95	0.73	112.60	0.02	0.04
4	Busy	215.22	0.49	61.93	0.01	0.04	Routine	221.11	0.47	33.90	0.01	0.03
5	Routine	200.66	0.51	70.99	0.01	0.04	Skincare	191.71	0.55	43.58	0.01	0.04
6	Industry	142.08	0.41	20.64	0.01	0.04	Coronavirus	191.71	0.49	31.99	0.01	0.03
7	Natural	140.15	0.39	31.14	0.01	0.03	Cosmetic	153.08	0.55	70.70	0.01	0.03
8	Fashion	138.77	0.37	37.96	0.01	0.03	Trick	149.65	0.18	5.49	0.01	0.01
9	Trick	133.73	0.20	6.72	0.01	0.02	Industry	148.49	0.43	29.19	0.01	0.03
10	Skincare	132.91	0.45	43.51	0.01	0.03	Company	144.68	0.29	3.78	0.01	0.02
11	Amazon	117.15	0.33	12.81	0.01	0.03	Video	144.15	0.31	41.56	0.01	0.02
12	Cosmetic	107.88	0.41	26.22	0.01	0.03	Salon	140.05	0.29	8.76	0.01	0.02
13	Celebrity	104.48	0.35	33.98	0.01	0.03	Instagram	139.25	0.33	23.16	0.01	0.02
14	Package	92.69	0.27	9.44	0.01	0.02	Natural	135.38	0.37	19.68	0.01	0.02
15	Plastic	88.66	0.29	16.95	0.01	0.03	Personal	126.34	0.35	10.90	0.01	0.03
16	Kardashian	88.52	0.12	1.13	0.01	0.01	Sleep	124.79	0.04	0.39	0.01	0.00
17	Company	86.08	0.29	4.53	0.01	0.03	Fashion	116.04	0.39	18.90	0.01	0.03
18	YouTube	82.33	0.16	1.97	0.01	0.02	Selena	112.04	0.18	4.64	0.01	0.01
19	Artist	79.31	0.20	2.39	0.01	0.02	Kardashian	109.73	0.27	5.10	0.01	0.02
20	Heal	77.88	0.20	11.42	0.01	0.02	Care	108.05	0.37	16.34	0.01	0.02
21	Professional	76.56	0.27	4.79	0.01	0.03	Trend	104.95	0.33	10.76	0.01	0.02
22	Tutorial	76.56	0.18	8.66	0.01	0.01	Store	100.76	0.37	10.11	0.01	0.03
23	Selena	75.12	0.18	3.95	0.01	0.02	Heal	100.76	0.29	7.39	0.01	0.02
24	Online	72.54	0.24	7.24	0.01	0.02	Pandemic	85.51	0.35	17.06	0.01	0.02
25	Rihanna	70.97	0.14	4.55	0.01	0.01	Change	85.51	0.37	15.39	0.01	0.02
26	Sephora	69.88	0.12	0.37	0.01	0.01	Global	83.40	0.35	11.58	0.01	0.03
27	Lipstick	69.44	0.29	8.10	0.01	0.03	Skin	79.81	0.20	2.01	0.01	0.02
28	Treatment	69.44	0.14	4.53	0.01	0.01	Palette	74.01	0.06	0.00	0.01	0.00
29	Palette	66.53	0.16	3.77	0.01	0.01	Lifestyle	70.42	0.22	2.02	0.01	0.02
30	Night	66.53	0.16	3.49	0.01	0.01	Home	65.72	0.18	1.36	0.01	0.01

^a^, ^b^, ^c^, ^d^Degree, betweenness, closeness, and eigenvector centrality, respectively

Before the COVID-19 pandemic, keywords such as “makeup” (_*Tf-idf*_ = 369.68), “product” (315.68), “brand” (276.77), “busy” (215.22), “routine” (200.66), “industry” (142.08), “natural” (140.15), “fashion” (138.77), “trick” (133.73), and “skincare” (132.91) appeared in the upper ranks of consumers’ awareness. Although keywords such as “product” (410.57), “makeup” (389.54), “brand” (328.95), “routine” (221.11), and “skincare” (191.71) appeared in the upper ranks, keywords such as “coronavirus” (191.71) and “pandemic” (85.51) appeared in the infectious disease keywords. These results indicate that a pandemic impacts general consumers’ awareness of cosmetics. Makeup remains the most significant product that composes consumers’ cosmetic awareness, irrespective of the pandemic situation. In the case of skincare, there has been an increase in rank since the pandemic.

### Analysis of network structure and centrality

**Fig. 2:**
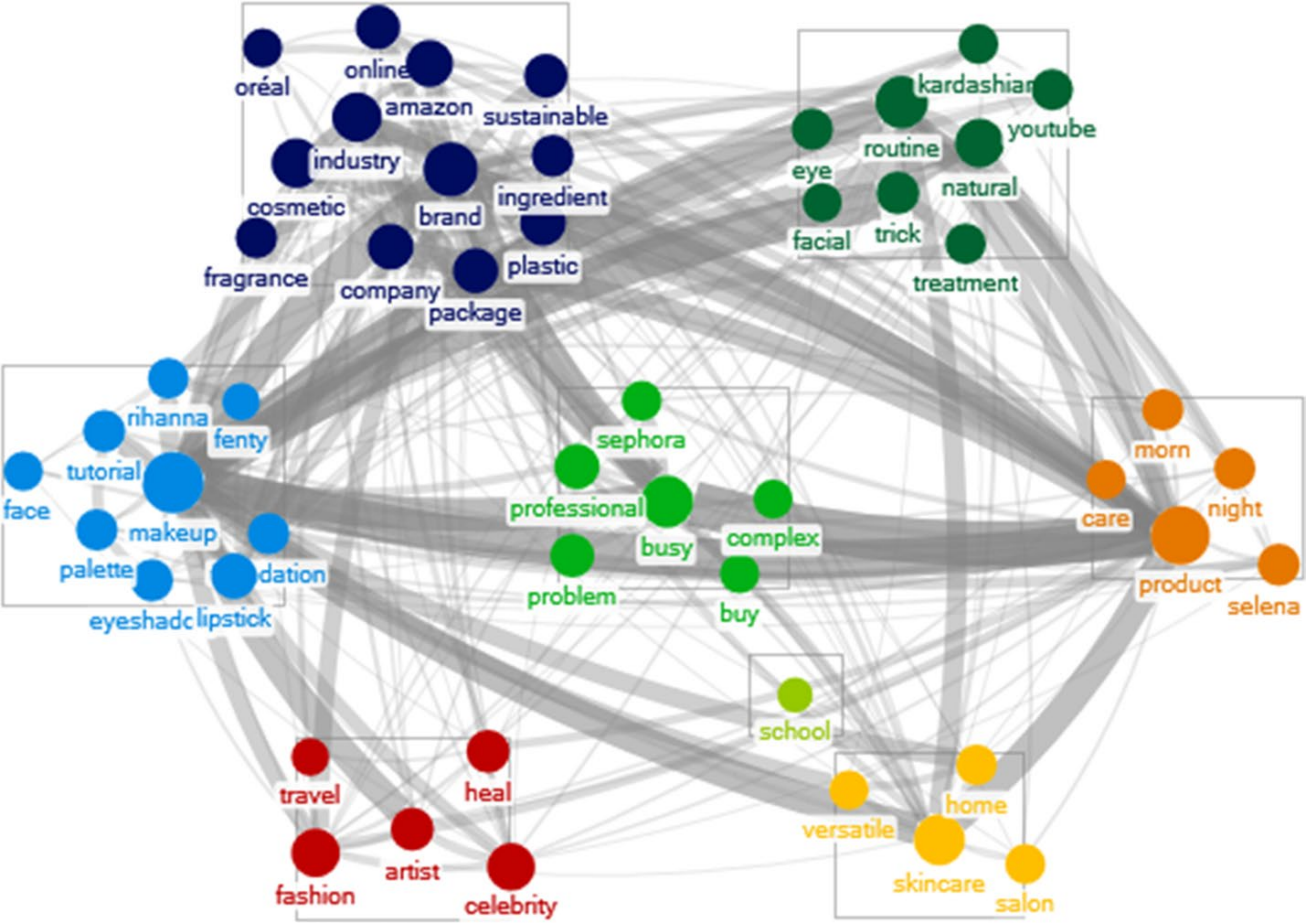
Consumers’ cosmetic awareness before COVID-19 (Wakita-Tsurumi algorithm)

**Fig. 3:**
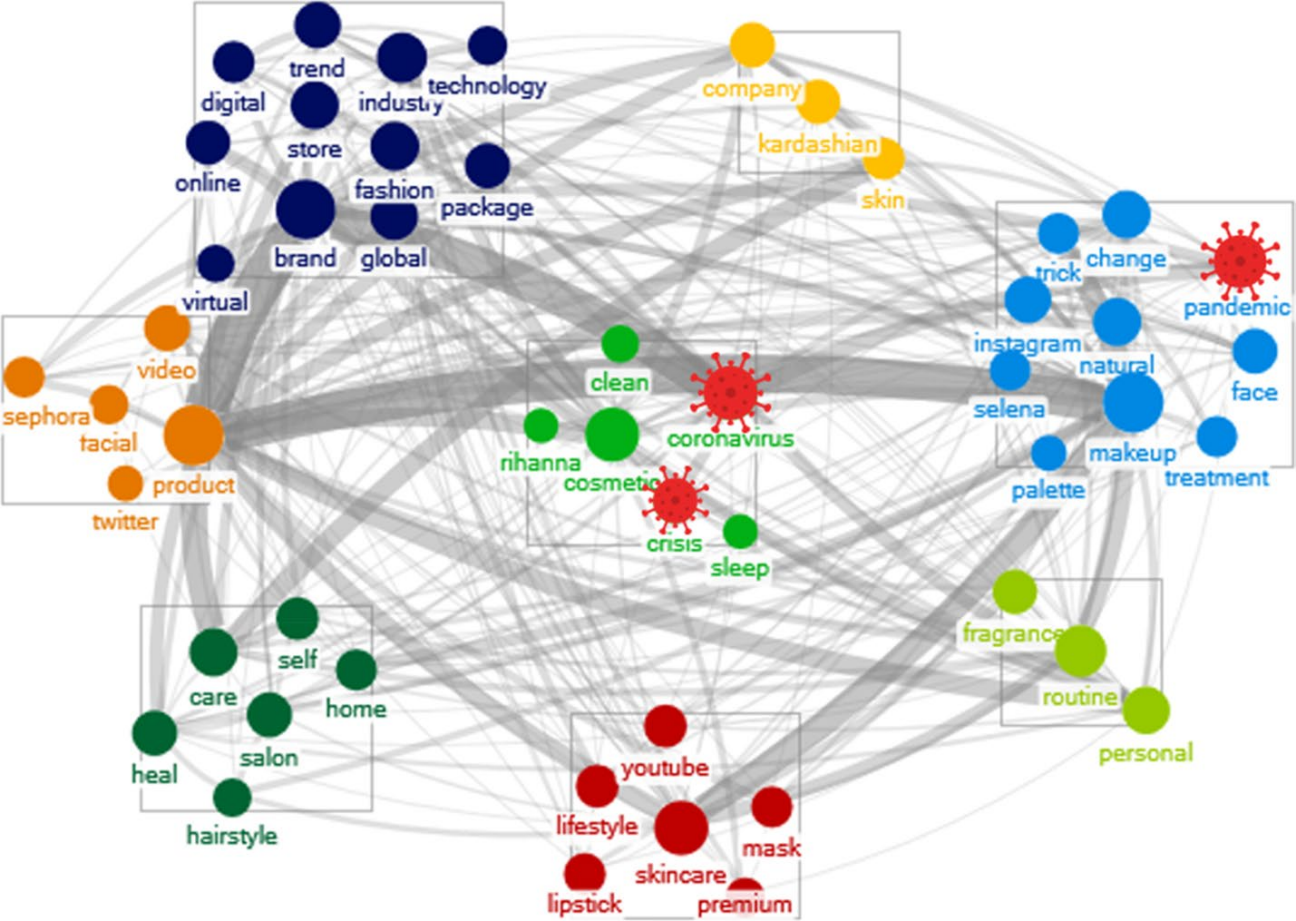
Consumers’ cosmetic awareness after COVID-19 (Wakita-Tsurumi algorithm)

We extracted the top 50 keywords based on the co-occurrence frequency and then clustered them using the Wakita-Tsurumi algorithm. Consumer awareness of  cosmetic behavior before COVID-19 outbreak  is illustrated in Fig. 2. Figure 3 represents  the beauty behavior after the COVID-19 outbreak. In Fig. 3, new keywords in relation to COVID-19 have emerged after the outbreak. The comparison of the network size is as follows. The maximum geodesic distance (diameter) of the networks for 2019 and 2020 is 3, and the overall networks were similar in size because both were equally limited to the top 50 keywords. In 2019, the total number of edges of the network was 590, the average geodesic distance was 1.76, and the graph density was 0.24. The total number of edges for 2020 was 706, the average geodesic distance was 1.72, and the graph density was 0.28. Compared to 2019, the significant increase in the number of edges in 2020 means that the correlation between keywords has increased within the context. As a result, we can see that the average geodesic distance of the graph has decreased, and the graph density has increased.

For centrality measurement, in the case of makeup, both degree and betweenness centrality declined in consumers’ cosmetic awareness after, rather than before, the pandemic. On the other hand, skincare showed an increase in all centrality items (C_d_, C_b_, and C_e_) except closeness centrality after the outbreak. On this basis, while the influence of makeup products has decreased, that of skincare products has increased. Moreover, in the results of *Tf-idf* and centrality measurement, keywords of color cosmetics, such as “lipstick,” “foundation,” and “eyeshadow,” which appeared as representative keywords, did not appear in the upper ranks after the outbreak.

Before COVID-19, skincare was classified in the compact groups such as “salon,” “versatile,” and “home,” but after the outbreak, skincare joined larger groups comprising “global,” “digital,” “clean,” and “technology,” which account for 28% of the whole group. Furthermore, before the pandemic, makeup was classified into groups containing the words “celebrity,” “fashion,” “Fenty,” and “tutorial.” After the pandemic, it was reclassified into groups containing “pandemic,” “change,” “face,” “treatment,” “skin,” and so on. During the pandemic, skincare was considered as cleansing to prevent coronavirus infection, and makeup was considered in terms of skin problems due to the pandemic situation.

According to the network analysis, the appearance of keywords related to coronavirus determined that pandemics affect consumers’ cosmetic awareness. After the outbreak, global consumers recognized skincare as extremely important. Makeup was also regarded as being important; however, its importance importance showed a declining trend.

### Consumers’ awareness of skincare and makeup

The results of preliminary investigation revealed that skincare and makeup appear as the main cosmetic behaviors and products. We therefore conducted a comparison of consumers’ awareness based on the keywords before and after pandemic (Table 3). After the outbreak, “coronavirus” appeared as an upper ranked *Tf-idf* keyword in every cosmetic product. This highlights the fact that the COVID-19 pandemic impacts consumers’ cosmetic awareness.

**Table 3:** Consumers’ cosmetic awareness before and after COVID-19

No	Skincare				Makeup			
	Before COVID-19 outbreak		After COVID-19 outbreak		Before COVID-19 outbreak		After COVID-19 outbreak	
	Word	***Tf-idf***	Word	***Tf-idf***	Word	***Tf-idf***	Word	***Tf-idf***
1	Product	210.83	Product	251.07	Beauty	201.56	Artist	232.93
2	Beauty	195.48	Beauty	193.12	Product	121.91	Beauty	196.14
3	Care	193.56	Care	163.83	Brand	77.25	Product	144.38
4	Skin	189.36	Skin	147.48	Cosmetic	75.96	Brand	90.86
5	Ingredient	120.41	Dermatologist	126.93	Euphoria	71.43	Celebrity	84.31
6	Makeup	116.99	Makeup	110.57	Busy	62.81	Cosmetic	82.18
7	Busy	111.84	Ingredient	106.79	Fashion	59.61	*Coronavirus*	*66.14*
8	Brand	111.30	Brand	91.59	Celebrity	52.89	**Lipstick**	**60.95**
9	Benefit	90.85	Vitamin	84.75	Wedding	50.74	Care	59.27
10	Fashion	70.76	Age	73.55	**Lipstick**	**48.85**	Sephora	55.89
11	Celebrity	67.11	Cosmetic	68.25	YouTube	47.62	**Eyeliner**	**53.80**
12	Morning	60.40	Routine	60.83	Skincare	40.80	Fashion	52.22
13	Retinol	60.18	Moisturize	55.63	Brush	38.86	Skin	49.89
14	Age	58.29	Self	55.17	**Eyeliner**	**38.86**	Eyebrow	47.90
15	**Serum**	**56.61**	Massage	49.38	Vogue	38.63	**Foundation**	**45.26**
16	Cosmetic	54.65	*Mask*	*47.81*	Sunscreen	36.85	*Mask*	*43.98*
17	Dermatologist	54.43	*Prevention*	*47.50*	Selfie	36.84	Palette	42.89
18	Treatment	51.01	**Maskne**	**46.92**	Palette	35.21	Skincare	33.40
19	Moisturize	50.34	**Sunscreen**	**45.55**	**Foundation**	**35.07**	Mascara	32.80
20	Routine	49.53	**Cleansing**	**45.30**	YouTuber	34.54	*Lockdown*	*32.80*
21	Vitamin	48.90	**Acne**	**38.39**	Skin color	34.54	Brush	29.22
22	**Sunscreen**	**41.37**	Treatment	36.78	Color	31.30	Treatment	28.11
23	Color	41.37	Heal	36.78	Tutorial	30.06	Home	26.39
24	Fit	41.31	*Lockdown*	*36.75*	Sephora	27.63	Hairstylist	26.39
25	Expert	37.61	**Serum**	**31.43**	Powder	27.63	Fragrance	26.39
26	**Cream**	**37.61**	Sephora	29.86	Ingredient	25.90	*COVID-19*	*26.39*
27	Effect	35.63	Retinol	29.86	Skin	23.47	*Distance*	*26.39*
28	Facialist	33.85	**Cream**	**28.58**	Cleansing	23.03	*Maskne*	*25.45*
29	Dermatology	33.85	Haircare	26.93	Pigmentation	23.03	Washing	25.05
30	**Cleansing**	**24.29**	*Coronavirus*	*26.93*	Eyeshadow	21.59	Wedding	25.05

Words in bold = major cosmetic products. Italics = COVID-19 related words

The results of comparing representative keywords of individual cosmetic products before and after the pandemic are shown below. First, in the case of skincare, “self” (_*Tf-idf*_ = 55.17), “mask” (47.81), “prevention” (47.50), “maskne” (46.92), “acne” (38.39), and “lockdown” (36.75) appeared as the representative keywords. In terms of consumer’s cosmetic awareness, this indicates the appearance of skin problems (or acne) caused due to wearing masks to prevent infection and the phenomena of self-care at home due to lockdown. The main products are serum (56.61/31.43), sunscreen (41.37/45.55), cream (37.61/28.58), and cleansers (24.29/45.30). Additionally, keywords such as “mask” (47.81) and “Maskne” (46.92) appear as representative keywords in cosmetic awareness, include skincare products for “acne” (38.39).

Second, in terms of consumers’ awareness on makeup, keywords such as “mask” (43.98), “lockdown” (32.80), “home” (26.39), “COVID-19” (26.39), “distance” (26.39), “maskne” (25.05), and “washing” (25.05) appeared after the outbreak. As with skincare, skin problems caused by wearing masks and cleansing the face appeared as the main interest after the spread of the disease. In addition, words related to social distancing and lockdown phenomena related to makeup started to appear. The representative products are “lipstick” (48.85/60.95), “eyeliner” (38.86/ 53.80), and “foundation” (35.07/45.26).

The analysis of consumers’ cosmetic awareness of skincare and makeup products before and after the outbreak of the COVID-19 pandemic revealed keywords related to infectious disease (coronavirus, mask, lockdown). Therefore, it can be argued that consumers’ awareness regarding individual cosmetic products has been affected by the pandemic.

### Spread of the pandemic and consumers’ interest in cosmetic products

#### Model evaluation

Since the confirmed cases of COVID-19 and search volume of individual cosmetic products’ keywords are measured with a single measurement object, significance and suitability, such as convergent validity, multi-collinearity, outer weights, and outer loading do not need to be judged. The PLS-SEM model is assessed by *R*^*2*^*, f*^*2*^*,* and *Q*^*2*^ values (Shin, 2018). In investigation of consumer behaviors, if the coefficient of determination for the endogenous variable is larger than *R*^*2*^ = 0.20, it is judged to have a very high estimated suitability (Hair et al., 2017). The values of *R*^*2*^ in the endogenous variable are skincare products (*R*^*2*^_serum_ = 0.31, *R*^*2*^_sunscreen_ = 0.21, *R*^*2*^_acne_ = 0.32, *R*^*2*^_cleanser_ = 0.15, and *R*^*2*^_cream_ = 0.25), makeup products (*R*^*2*^_eyeliner_ = 0.21, *R*^*2*^_foundation_ = 0.36, and *R*^*2*^_lipstick_ = 0.27), and mask (*R*^*2*^ = 0.20). The values of *R*^*2*^ for acne, serum, and foundation are larger than *R*^*2*^ = 0.30. The majority of the other endogenous variables were larger than *R*^*2*^ = 0.20, indicating a high level of suitability.

If the effect size (*f*^*2*^) in PLS-SEM is greater than *f*^*2*^ = 0.02, it indicates a small effect size for latent exogenous variables on latent endogenous variables. If it is greater than *f*^*2*^ = 0.15, it indicates a medium effect size. If it is greater than *f*^*2*^ = 0.35, it indicates a large effect size. The interest in cosmetic products due to the spread of disease showed foundation as (*f*^*2*^ = 0.53), which has an effect size larger than *f*^*2*^ = 0.35 and it was identified as having the largest contribution. Serum (*f*^*2*^ = 0.17), acne (*f*^*2*^ = 0.25), lipstick (*f*^*2*^ = 0.28), and mask (*f*^*2*^ = 0.25) have medium effect sizes. Sunscreen (*f*^*2*^ = 0.09), cleanser (*f*^*2*^ = 0.07), and eyeliner (*f*^*2*^ = 0.04) have small effect sizes. Masks, cream (*f*^*2*^ = 0.21), foundation (*f*^*2*^ = 0.24), and lipstick (*f*^*2*^ = 0.26) showed medium effect sizes, and the others showed small effect sizes (*f*^*2*^_serum_ = 0.08, *f*^*2*^_sunscreen_ = 0.05, *f*^*2*^_acne_ = 0.03, *f*^*2*^_cleansing_ = 0.03, and *f*^*2*^_eyeliner_ = 0.11). Next, by identifying the estimated suitability (*Q*^*2*^) through the blindfolding process, all *Q*^*2*^ values of the latent endogenous variables were larger than 0, indicating the suitability of the entire constitutive model.

### Hypothesis testing

#### SOP and interest in cosmetic products

**Fig. 4:**
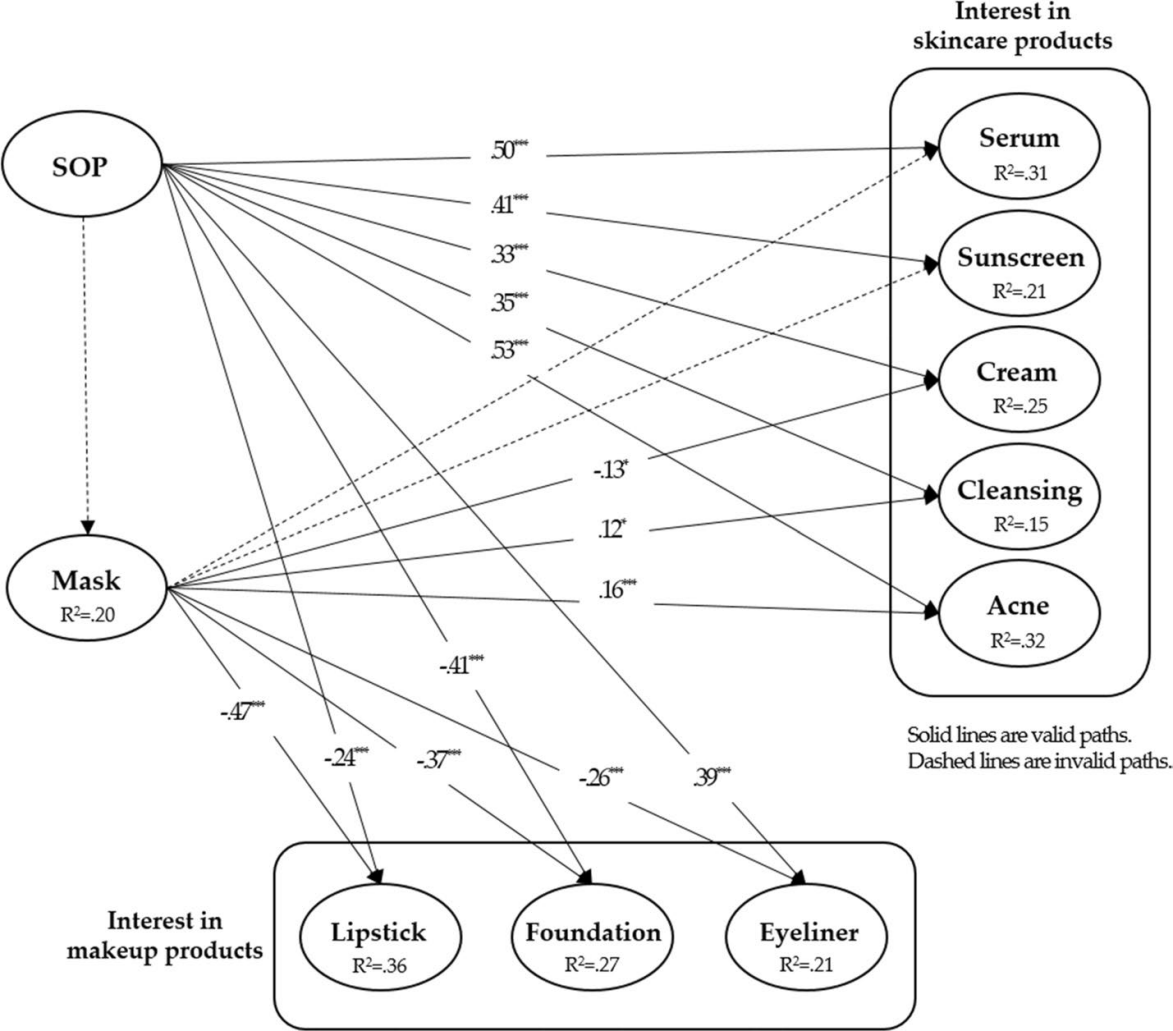
Results of partial least squares structural equation modeling (PLS-SEM)

The impact of the spread of the COVID-19 pandemic on global consumers’ awareness regarding individual cosmetic products is as follows (Table 4; Fig. 4). The SOP had a positively influencing relationship with all skincare products: serum (*β* = 0.50, *p* < 0.001), sunscreen (*β* = 0.40, *p* < 0.001), cream (*β* = 0.33, *p* < 0.001), cleanser (*β* = 0.34, *p* < 0.001), and acne (*β* = 0.52, *p* < 0.001). Therefore, it was clear that as the number of confirmed cases increased, global consumers’ interest in skincare products also increased. This result statistically proves news reports and interviews about changes in cosmetic behaviors due to the pandemic (Cerullo, 2020; Wallace & CNN Business, 2020). Therefore, H1 (H1a, H1b, H1c, H1d, H1e) was supported.

**Table 4:** Direct path coefficients of SOP and interest in mask and individual cosmetic products

	Direct path	Path coefficient	S.E	***t***	Result	Total
H1						
H1a	SOP → serum	0.50	0.04	13.65^***^	Accepted	Accepted
H1b	SOP → sunscreen	0.41	0.04	9.76^***^	Accepted	
H1c	SOP → cream	0.33	0.05	7.06^***^	Accepted	
H1d	SOP → cleansing	0.35	0.05	6.93^***^	Accepted	
H1e	SOP → acne	0.53	0.04	14.17^***^	Accepted	
H2						
H2a	SOP → lipstick	− 0.24	0.05	4.57^***^	Rejected	Partially accepted
H2b	SOP → foundation	− 0.41	0.05	8.54^***^	Rejected	
H2c	SOP → eyeliner	0.39	0.06	6.78^***^	Accepted	
H3	SOP → mask	0.11	0.06	1.89	Accepted	Accepted
H4						
H4a	Mask → serum	0.08	0.05	1.69	Rejected	Partially accepted
H4b	Mask → sunscreen	0.07	0.05	1.26	Rejected	
H4c	Mask → cream	− 0.13	0.05	2.38^*^	Rejected	
H4d	Mask → cleansing	0.12	0.05	2.40^*^	Accepted	
H4e	Mask → acne	0.16	0.04	3.55^***^	Accepted	
H5						
H5a	Mask → lipstick	− 0.47	0.04	10.82^***^	Accepted	Accepted
H5b	Mask → foundation	− 0.37	0.05	7.80^***^	Accepted	
H5c	Mask → eyeliner	− 0.26	0.05	4.94^***^	Accepted	

*SOP* spread of pandemic

^*^p < 0.05, ^**^p < 0.01, ^***^p < 0.001, respectively

The SOP had a negatively influencing relationship with lipstick (*β* =  − 0.23, *p* < 0.001) and foundation (*β* =  − 0.41, *p* < 0.001). This is statistical proof that the sales of makeup products have decreased (Cerullo, 2020; Halliday, 2020). This decrease is due to wearing masks or staying at home to prevent infection and spreading of the virus. Since people decide not to apply makeup to the area of the face covered by a mask does not need to have makeup applied, interest in makeup has naturally fallen. Meanwhile, eyeliner (*β* = 0.38, *p* < 0.001) showed a positively influencing relationship with regards to interest in the product. Opposite to the case of lipsticks and foundations, the eyes are still visible when wearing masks, so consumers tend to pay more attention to eye makeup, leading to the increased interest in eyeliners (see Table 4).

“Maskne” behavior manifests in that consumers tend to focus their efforts on the exposed area of their face. This is also similar to Muslim females’ cosmetic behavior, in which they pay more attention to eye makeup as they cover the rest of their face due to their religious ideology. In each case, the social setting forces people to hide their facial area, which leads to the limited application of makeup, that is, eye makeup. Hill et al. (2012) argued cosmetics such as lipsticks are a decorative product. In a pandemic situation, since a mask covers the nose, lips, and chin, eye makeup products are considered a decorative product. Therefore, H2c was accepted, and H2a and H2b were rejected.

### Interest in face mask and cosmetic products

The SOP and consumers’ interest in masks were *β* = 0.11, *p* > 0.05, indicating that it does not have a significantly influencing relationship; H3 is therefore rejected (Table 5). However, the result of dividing the confirmed cases of COVID-19 by the timing of the spread was interesting. In the early phase from January to April 2020, it presented a high coefficient (*β* = 0.80, *p* < 0.001). In the next phase from May to July 2020, SOP also revealed a positive impact on masks (*β* = 0.57, *p* < 0.001), but it was lower than the previous phase. However, since the pandemic has been prolonged, the interest in masks has fallen and it seems to have levelled off. Consequently, the increase of confirmed cases may have influenced the relationship with the interest in masks. However, as the pandemic continues, it does not have a significantly influencing relationship; H3 was therefore accepted.

**Table 5:** Direct path coefficients of SOP and interest in mask according to three phases of COVID-19 pandemic

	Path coefficient	S.E	*t*	*f*^*2*^	*Q*^*2*^
2020. 1. 21–2020. 4. 30	0.80	0.02	27.63***	1.88	0.65
2020. 5. 1–2020. 7. 31	0.57	0.06	9.177***	0.48	0.32
2020. 8. 1–2020. 9. 30	− 0.21	0.12	1.653	0.04	0.03

Classification: based on the overall data and the spread of COVID-19

Although the interest in face masks did not form significantly influencing relationships with serum, sunscreen, and cream, it showed a positively influencing relationship with acne (*β* = 0.15, *p* < 0.001) and cleansing (*β* = 0.11, *p* < 0.05). This is similar to the background of the appearance of the word “maskne,” which proves the interest in masks has a relationship with skin problems, such as acne, caused by wearing masks. On the other hand, cream (*β* =  − 0.12, *p* < 0.05) had a significantly negative influencing relationship. Since “cream” can include moisturizing cream, BB cream, sun cream, and so on, it can be assumed that this affected the result.

Therefore, H4a, H4b, and H4c were rejected, and H4d and H4e were accepted. Meanwhile, interest in masks showed a negatively influencing relationship with lipstick (*β* =  − 0.46, *p* < 0.001), foundation (*β* =  − 0.36, *p* < 0.001), and eyeliner (*β* =  − 0.25, *p* < 0.001). As consumers’ interest in masks increases, interest in makeup products for the entire face decreases. Therefore, H5 (H5a, H5b, H5c) was accepted.

## Conclusions

This research explored the changes in consumers’ awareness of cosmetic products during the pandemic. The results suggest that epidemiological situations, such as COVID-19, affect global consumers’ awareness of cosmetics and each cosmetic category (skincare and makeup). The SOP has increased consumers’ interest in skincare products, while interest in makeup products (lipstick and foundation) has decreased except for eyeliner products. This suggests that anxiety regarding of severity of COVID-19 not only affects interest in skin conditions but also that consumers seek to maintain their social self-esteem by wearing makeup on the exposed areas of their face (Gruenewald et al., 2004). However, interest in makeup products was shown to have a negative influencing relationship with masks. Obligation to wear a mask for an extended period may cause discomfort experiences (Park et al., 2020). In terms of masks and makeup products, consumers have expressed complaints and discussed the side effects of makeup and wearing PPE in the online community.

The SOP did not have a significantly influencing relationship with consumers’ interest in face masks. In the early stages of increased confirmed cases, a positively influencing relationship with the interest in face masks was observed, but it did not form a significantly influencing relationship as the pandemic progressed. It is obvious that the prolonged pandemic situation makes the preventive measure ineffective compared to the early phase (Bults et al., 2011). The outbreak has lasted more than a year and mask wearing has become the norm. Therefore, regardless of whether the number of confirmed cases is on the rise, the interest in mask wearing would not follow a similar trend. A summary of the results of this study is shown in (Table 6).

**Table 6:** Summary of changes in consumers’ cosmetic awareness and interest after COVID-19 outbreak

Method	Key results
Consumers’ awareness	
Skincare	Skincare products has increased in consumers’ beauty awareness Skincare was considered as cleansing to prevent coronavirus infection Skin problems caused due to wearing masks to prevent infection and the phenomena of self-care at home
Makeup	Makeup products has decreased in consumers’ beauty awareness Keywords of color cosmetics did not appear in the priority list in consumers’ beauty awareness Makeup was considered in terms of skin problems due to the pandemic situation As with skincare, skin problems caused by wearing masks and cleansing the face appeared as the main interest
Consumers’ interest	
Skincare	The spread of pandemic had a positively influencing relationship with skincare products (e.g. serum, sunscreen, cream, cleanser, and acne) Interest in face mask had a positively influencing relationship with acne and cleansing
Makeup	The spread of pandemic had a negatively influencing relationship with lipstick and foundation Interest in face mask had a negatively influencing relationship with lipstick, foundation, and eyeliner

In terms of practical contribution, this study analyzed global consumers’ macro awareness, allowing cosmetic companies to examine comprehensive opinions about consumers’ beauty habits during a pandemic. Therefore, if future pandemics have similar characteristics to the current COVID-19 outbreak, beauty companies have to pay more attention to the production of sanitary, cleanliness, and so-called trouble-care products. As consumers also consider their social self and social self-esteem as important as skincare despite the spread of pandemic, it is necessary to establish a niche makeup strategy to enable consumers to represent their own identity.

This study has the following limitations. We collected data in English, which is the global official language; therefore, it is difficult to apply this study to non-English speaking countries. Newspaper articles and industrial reports were used as references to best measure the social phenomena occurring in real time, and macro data were used to encompass all consumers. Future research should be conducted with much more elaborate theories and accurate measurements. From another viewpoint, demand for cosmetic products decreased in the early stage of the outbreak, and people created new forms of beauty behaviors such as “stay-home makeup” and “makeup for Zoom meetings.” Moreover, consumers’ preferences or behaviors regarding cosmetic products are subject to change, depending on the characteristics of infectious diseases. For example, if a new pandemic is not caused by a respiratory infection, the results derived from this study cannot be applied. Subsequent studies need to analyze new beauty behaviors after COVID-19.

Despite the aforementioned limitations, this study is considered important as it has statistically demonstrated that in a pandemic situation, interest in cosmetics may vary depending on their function and category. In addition, the motivations for wearing makeup could be construed as behavior of maintaining the social self. This provides the cosmetic industry some insights into the fact that consumers’ concerns about appearance in pandemic situations are diverse. Consumers are diverse and every case has customized needs for managing their societal appearance. Thus, the results have some managerial implications for the cosmetic industry in terms of which products they should focus on for production and establishing marketing strategies during pandemic situations. Moreover, as a new approach, this study expanded the generalizable scope by using macroscopic data such as social media text and volume of Google keyword searches.

## Data Availability

Please contact author for data requests.
